# Relationship of Having Hobbies and a Purpose in Life With Mortality, Activities of Daily Living, and Instrumental Activities of Daily Living Among Community-Dwelling Elderly Adults

**DOI:** 10.2188/jea.JE20150153

**Published:** 2016-07-05

**Authors:** Kimiko Tomioka, Norio Kurumatani, Hiroshi Hosoi

**Affiliations:** Nara Prefectural Health Research Center, Nara Medical University, Kashihara, Nara, Japan

**Keywords:** mortality, activities of daily living, elderly, hobbies, purpose in life

## Abstract

**Background:**

This study’s aim was to clarify the relationship of having hobbies and a purpose in life (PIL; in Japanese, *ikigai*) with mortality and a decline in the activities of daily living (ADL) and instrumental ADL (IADL) among the community-dwelling elderly.

**Methods:**

Prospective observational data from residents aged ≥65 years who were at increased risk for death (*n* = 1853) and developing a decline in ADL (*n* = 1254) and IADL (*n* = 1162) were analyzed. Cox proportional hazard models were used for mortality analysis of data from February 2011 to November 2014. ADL and IADL were evaluated using the Barthel Index and the Tokyo Metropolitan Institute of Gerontology Index of Competence, respectively. ADL and IADL were assessed at baseline and follow-up and were evaluated using logistic regression models. Fully adjusted models included terms for age, gender, BMI, income, alcohol intake, smoking history, number of chronic diseases, cognitive function, and depression.

**Results:**

During the follow-up of eligible participants, 248 had died, 119 saw a decline in ADL, and 178 saw a decline in IADL. In fully adjusted models, having neither hobbies nor PIL was significantly associated with an increased risk of mortality (hazard ratio 2.08; 95% confidence interval [CI], 1.47–2.94), decline in ADL (odds ratio 2.74; 95% CI, 1.44–5.21), and decline in IADL (odds ratio 1.89; 95% CI, 1.01–3.55) compared to having both hobbies and PIL.

**Conclusions:**

Although effect modifications by cognitive functioning and depression cannot be ruled out, our findings suggest that having hobbies and PIL may extend not only longevity, but also healthy life expectancy among community-dwelling older adults.

## INTRODUCTION

Not only behavioral factors but also psychological factors have serious effects on mortality risk in the elderly. Prior studies have reported that behavioral factors, such as smoking,^1^ excessive alcohol consumption,^2^ and physical inactivity,^2^^,^^3^ and psychological factors, such as a low subjective sense of well-being,^4^ hopelessness,^5^ loneliness,^6^ and dissatisfaction,^7^ were associated with an increased risk of mortality. Additionally, behavioral factors, such as hobbies, and psychological factors, such as purpose in life (PIL), are considered important components of successful aging.^8^ Prior studies of community-dwelling elderly adults have reported that high engagement in hobbies was associated with significantly decreased mortality,^9^^–^^11^ and that a lack of PIL was significantly associated with an increased risk of mortality.^12^^,^^13^ Although these above-mentioned previous studies suggest that a lack of hobbies and PIL can have grave consequences in older adults, from the early prevention viewpoint, it’s also important to see what factors can directly affect an elderly person’s ability to live independently. Activities of daily living (ADL) and instrumental activities of daily living (IADL) are suitable indicators for these outcomes.^14^ However, to our knowledge, no studies have been conducted on the relationship of having hobbies and PIL with ADL and IADL among the community-dwelling elderly. Since social participation, including hobby activities and having PIL, are potentially modifiable factors,^15^ clarifying the association between hobbies, PIL, and functional capacity can provide a better understanding of how to increase the healthy life years of the elderly.

Our study hypothesis is that community-dwelling elderly who have neither hobbies nor PIL are more likely to experience not only an increased risk of mortality but also a decline in their ability to perform ADL or IADL compared with those who have both hobbies and PIL.

## METHODS

### Study area and subjects

The target area for this study was Shimoichi Town in Nara Prefecture, a rural Japanese town with an approximate population of 6900 residents. The target population was all residents aged 65 years and older. In February 2011, the town office mailed the baseline questionnaires to 2481 community-dwelling older adults (response rate: 83.1%). Figure displays the flow diagram of the enrollment of study participants. Among the 2061 persons (858 males and 1203 females) who participated in the baseline survey, we excluded 208 because of invalid responses for ADL, IADL, hobbies, and/or PIL. Thus, 1853 valid responses were obtained; mortality follow-up of these 1853 participants was performed for the period from February 2011 until November 2014. Compared to persons with valid responses, subjects without valid responses were marginally significantly older and fewer were males, but there was no difference in income (see eTable 1). Of the baseline population, 1556 were independent in their ADL, and 1399 had full ability to perform IADL. In July 2014, similar postal questionnaire surveys were sent out to obtain follow-up data. After excluding individuals with missing follow-up scores (ie, persons who had died, moved out of the study area, had invalid follow-up data for ADL/IADL, or did not respond to the follow-up survey), 1254 (80.6% of 1556) were analyzed for ADL, and 1162 (83.1% of 1399) were analyzed for IADL. Subjects excluded from the follow-up study were significantly older and had lower incomes than analyzed participants (see eTable 1).

**Figure.**
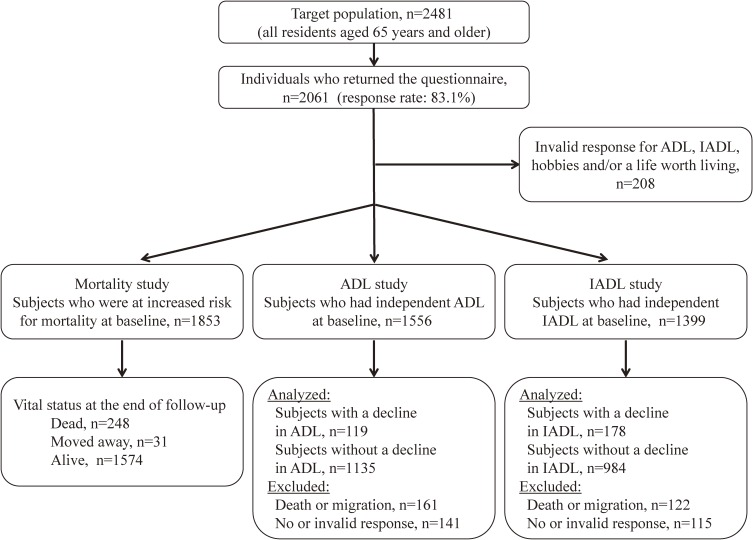
Flow diagram of the enrollment of study participants. ADL, activities of daily living; IADL, instrumental activities of daily living.

All study participants provided signed informed consent. This study protocol was approved by the Nara Medical University Ethics Committee (approval number 991).

### Assessment of outcomes

The outcomes of interest in this study were all-cause mortality, ADL decline, and IADL decline. Regarding mortality, information about death and migration was obtained from the Shimoichi Town Hall; the vital statuses of the study participants as of the end of November 2014 were determined through their residential registration cards and death certificates. ADL was evaluated using the Barthel index (score range 0–100)^16^; higher scores indicate better ADL. Subjects with ADL scores ≥90 were considered independent.^17^ Decline in ADL was defined as a change from a score of ≥90 at baseline to a score of <90 at follow-up. Thus, participants with independent ADL at baseline were divided into two groups according to ADL score at follow-up: decline (<90) and no decline (≥90). IADL was determined using the 5-item Tokyo Metropolitan Institute of Gerontology (TMIG) Index of Competence (ie, the ability to use public transportation, daily shopping for necessities, meal preparation, bill paying, and managing their bank deposits).^18^ A full score of 5 was categorized as independent, and a score of 0–4 was categorized as dependent.^19^ Decline in IADL was defined as a change from a maximum score at baseline to anything less than a maximum score at follow-up. Thus, participants with a maximum baseline score on IADL were divided into two groups according to the IADL score at follow-up: decline (<5) and no decline (5 points).

### Assessment of hobbies and a purpose in life (PIL)

For assessing hobbies and PIL, the questionnaire asked: “Do you have any hobbies?” and “Do you have *ikigai*?’’ directly translated as ‘‘Do you have a reason for living?’’. *Ikigai* is generally meant as feeling that life has a purpose, be it specific to the person themselves or overall, and can be briefly translated into either having a “purpose in life” (overall) or a “reason for living” (specific).^13^^,^^20^ Therefore, in this study, we treated *ikigai* as PIL. The response to each question was simply ‘yes’ or ‘no’. Subjects who answered ‘yes’ to the questions were defined as persons who have hobbies/PIL. The study participants were classified into four groups according to their responses to these two questions: both hobbies and PIL, hobbies only (ie, subjects who had hobbies but not PIL), PIL only (ie, subjects who had PIL but not hobbies), and neither hobbies nor PIL.

### Covariates

Based on previous studies,^9^^–^^13^^,^^21^^–^^24^ age, gender, income, body mass index (BMI), alcohol consumption, smoking habits, self-reported medical conditions, cognitive functioning, and depression were used as covariates that might correlate with hobbies, PIL, and mortality/disability. Information on age, gender, and income was obtained from the municipal office, and other covariates were gleaned from a questionnaire.

Income was used as an indicator of socioeconomic status. Income was classified into low (annual household income <1 million yen), medium (annual household income ≥1 million yen but annual individual income <1 million yen), and high (annual income of the subject ≥1 million yen). BMI was calculated as weight (kg) divided by the square of height in meters (m^2^), and defined as underweight (<18.5 kg/m^2^), normal (18.5–<25.0 kg/m^2^), and overweight (≥25.0 kg/m^2^). For lifestyle habits, the subjects were asked about alcohol consumption (nondrinkers, social drinkers, occasional drinkers, or daily drinker) and smoking status (never, former, or current). For medical conditions, the subjects were asked whether they were under medical treatment for any of seven major chronic conditions (hypertension, musculoskeletal disease, heart disease, diabetes mellitus, stroke, dyslipidemia, and cancer). The Cognitive Performance Scale (CPS: score range of 0 to 6) was used to rate cognitive functioning.^25^ A score of ≥1 was felt to be evidence of lower cognitive functioning. Depression ratings were determined through the use of the Geriatric Depression Scale (GDS-5: score range 0–5),^26^ a 5-item form. Depression was considered a problem if a score of ≥2 was found.

All covariates were dichotomized: age (65–74 vs ≥75 years), gender (male vs female), income (low vs medium or high), BMI (normal vs underweight or overweight), alcohol intake (daily drinkers or not), smoking (former or current smokers vs never-smokers), the number of chronic diseases (≥2 or not), cognitive functioning (CPS score ≥1 or not), and depression (GDS-5 score ≥2 or not).

### Statistical analysis

For mortality analysis, survival times were calculated starting from the enrollment date to either the date of death, the follow-up date (end of November 2014), or the latest registration date. Subjects who had relocated during the follow-up period were given censored survival times. Censored survival times were also given to cohort members who remained town residents when the follow-up finished.^27^ The Cox proportional hazards model was used to evaluate the univariate and multivariate relations between hobbies, PIL, and mortality. For disability analysis, logistic regression models were used to analyze whether hobbies and PIL at baseline were associated with a decline in ADL or IADL at 42-month follow-up. The independent variable was category of hobbies and PIL at baseline. In each model, the category of participants who had both hobbies and PIL was set as the referent category. For analysis of the association of hobbies and PIL with mortality, ADL, and IADL, we first conducted univariate analyses (Model 1) and then adjusted it for age and gender (Model 2). Subsequently, in addition to the variables adjusted in Model 2, income, BMI, alcohol intake, smoking history, and the number of chronic diseases were included in the model (Model 3). Finally, in addition to the variables adjusted in Model 3, cognitive functioning and depression were included in the model (Model 4). Additional stratified analyses by age, income, cognitive functioning, and depression were conducted to confirm whether these factors modified the effect of hobbies and PIL on mortality, ADL, and IADL. The level of significance was 0.05 (two tailed). Statistical analyses were performed using SPSS (version 17.0; SPSS Japan Inc., Tokyo, Japan).

## RESULTS

Of the 1853 subjects (42.3% males, mean age 76.4 [standard deviation {SD} 7.1] years) analyzed, 62.4% (*n* = 1156) had hobbies and PIL, 7.4% (*n* = 137) had hobbies only, 16.4% (*n* = 303) had PIL only, and 13.9% (*n* = 257) had neither hobbies nor PIL. Baseline characteristics of study participants according to hobbies and PIL are shown in Table 1. Compared with those who had both hobbies and PIL, those who had neither hobbies nor PIL were more likely to be older, suffer from depression, and have low income, abnormal BMI, temperance in drinking, a recent medical history of stroke, and poor cognitive functioning. There were no marked differences among the four groups in gender, smoking history, and prevalence of chronic diseases other than stroke.

**Table 1.** Baseline characteristics of study participants according to hobbies and a purpose in life (*ikigai*)

Characteristics	Both		Hobbies only		Purpose in life only		Neither		*P*^a^
	*n* = 1156		*n* = 137		*n* = 303		*n* = 257		
			
	*n*	%	*n*	%	*n*	%	*n*	%	
Demographics									
Age, years									
≥75	557	48.2	87	63.5	209	69.0	203	79.0	<0.001
Gender									
Male	505	43.7	56	40.9	111	36.6	111	43.2	0.162
Income									
Low	404	34.9	64	46.7	152	50.2	149	58.0	<0.001
Body mass index									
Normal (18.5–25.0 kg/m^2^)	752	65.1	86	62.8	186	61.4	120	46.7	<0.001
Lifestyle habits									
Alcohol intake									
Daily drinker	208	18.0	24	17.5	39	12.9	27	10.5	0.007
Smoking history									
Ex- or current smokers	411	35.6	49	35.8	86	28.4	90	35.0	0.123
Physical and mental conditions									
Chronic diseases under medical treatment									
Hypertension	490	42.4	54	39.4	139	45.9	97	37.7	0.234
Musculoskeletal disease	181	15.7	29	21.2	55	18.2	52	20.2	0.132
Heart disease	142	12.3	16	11.7	51	16.8	43	16.7	0.073
Diabetes mellitus	144	12.5	15	10.9	41	13.5	31	12.1	0.902
Stroke	45	3.9	11	8.0	23	7.6	37	14.4	<0.001
Dyslipidemia	80	6.9	8	5.8	12	4.0	10	3.9	0.112
Cancer	32	2.8	4	2.9	8	2.6	12	4.7	0.425
The number of chronic diseases under medical treatment									
≥2	282	24.4	34	24.8	87	28.7	75	29.2	0.241
Cognitive functioning (Cognitive Performance Scale)									
Poor (score ≥1)	208	18.0	61	44.5	119	39.3	185	72.0	<0.001
Depression (Geriatric Depression Scale)									
Depression (score ≥2)	251	21.7	71	51.8	118	38.9	190	73.9	<0.001

^a^Differences between the 4 groups were analyzed using Fisher’s exact test.

Follow-up for mortality could be completed for 1822 of the 1853 subjects enrolled (1574 alive and 248 deceased), because 31 had relocated away from Shimoichi Town by the time of follow-up (Figure). Table 2 shows the results from the Cox proportional hazards models of the association between having hobbies, having PIL, and mortality. In all models, having PIL only as well as having neither hobbies nor PIL were significantly associated with an increased risk of mortality. In Model 4, where the data was adjusted for all covariates, the hazard ratios (HRs) of mortality were 1.66 (95% confidence interval [CI], 1.17–2.35) for those who had PIL only and 2.08 (95% CI, 1.47–2.94) for those who had neither hobbies nor PIL compared with those who had both hobbies and PIL. Moreover, in additional analysis that excluded the deaths that occurred within the first 11 months of follow-up, these associations remained statistically significant: HR 1.65 (95% CI, 1.12–2.42) for those who had PIL only, and HR 1.95 (95% CI, 1.31–2.89) for those who had neither hobbies nor PIL.

**Table 2.** Results from cox proportional hazards models of association between hobbies, a purpose in life (*ikigai*), and mortality (*n* = 1853)

	Number of deaths	Person-years	Rate^a^	Crude HR (95% CI)	Adjusted HR (95% CI)		
	
				Model 1	Model 2	Model 3	Model 4
Both	91	4184.7	2.17	1.00	1.00	1.00	1.00
Hobbies only	18	469.8	3.83	1.77 (1.07–2.93)	1.58 (0.95–2.61)	1.53 (0.92–2.54)	1.14 (0.68–1.91)
Purpose in life only	56	1025.9	5.46	2.51 (1.80–3.50)	2.16 (1.55–3.03)	2.03 (1.44–2.85)	1.66 (1.17–2.35)
Neither	83	782.0	10.61	4.87 (3.62–6.56)	3.82 (2.82–5.17)	3.37 (2.47-4.59)	2.08 (1.47–2.94)

CI, confidence interval; HR, hazard ratio.

^a^Mortality rate per 100 person-years.

Model 1: Unadjusted.

Model 2: Adjusted for age and gender.

Model 3: Adjusted for age, gender, income, BMI, alcohol intake, smoking, and the number of chronic diseases.

Model 4: In addition to Model 3, cognitive functioning and depression were included.

Table 3 shows the results from logistic regression models of the association between having hobbies, having PIL, and a decline in ADL or IADL. Of the 1254 subjects with an independent baseline ADL and valid follow-up scores, 916 had both hobbies and PIL, 81 had hobbies only, 178 had PIL only, and 79 had neither hobbies nor PIL. During the 42-month follow-up, new ADL decline developed in 119 participants: 53 (5.8%) of those with both hobbies and PIL, 13 (16.0%) of those with hobbies only, 32 (18.0%) of those with PIL only, and 21 (26.6%) of those with neither hobbies nor PIL. In the crude model without any covariate adjustments (Model 1), hobbies only, PIL only, and neither hobbies nor PIL were strongly associated with a decline in ADL. However, after adjusting for all covariates, including cognitive functioning and depression (Model 4), the association of having hobbies only with ADL decline disappeared, and significant associations remained for having PIL only and having neither hobbies nor PIL: OR 1.95 (95% CI, 0.96–3.95) for those who had hobbies only, OR 2.56 (95% CI, 1.55–4.22) for those who had PIL only, and OR 2.74 (95% CI, 1.44–5.21) for those who had neither hobbies nor PIL compared with those who had both hobbies and PIL. Of the 1162 subjects with independent baseline IADL and valid follow-up scores, 870 had both hobbies and PIL, 71 had hobbies only, 159 had PIL only, and 62 had neither hobbies nor PIL. Further, 42-month declines in IADL were observed in 178 participants: 113 (13.0%) of those with both hobbies and PIL, 9 (12.7%) of those with hobbies only, 36 (22.6%) of those with PIL only, and 20 (32.3%) of those with neither hobbies nor PIL. In the univariate logistic regression analyses (Model 1), having PIL only as well as having neither hobbies nor PIL were significantly associated with a decline in IADL. After adjusting for all covariates (Model 4), these associations were attenuated but remained significant: OR 1.58 (95% CI, 1.02–2.47) for those who had PIL only and OR 1.89 (95% CI, 1.01–3.55) for those who had neither hobbies nor PIL compared with those who had both hobbies and PIL.

**Table 3.** Results from logistic regression models of association between hobbies, a purpose in life (*ikigai*), and a decline in ADL or IADL

Outcome	Outcome^a^/Total,^b^ *n*/*n* (%)	Crude OR (95% CI)	Adjusted OR (95% CI)		
	
		Model 1	Model 2	Model 3	Model 4
**Decline in ADL (*n* = 1254)**					
Both	53/916 (5.8)	1.00	1.00	1.00	1.00
Hobbies only	13/81 (16.0)	3.11 (1.62–5.99)	2.66 (1.36–5.19)	2.52 (1.28–4.97)	1.95 (0.96–3.95)
Purpose in life only	32/178 (18.0)	3.57 (2.23–5.73)	3.04 (1.88–4.92)	2.93 (1.79–4.79)	2.56 (1.55–4.22)
Neither	21/79 (26.6)	5.90 (3.33–10.44)	4.81 (2.68–8.65)	4.30 (2.36–7.82)	2.74 (1.44–5.21)
**Decline in IADL (*n* = 1162)**					
Both	113/870 (13.0)	1.00	1.00	1.00	1.00
Hobbies only	9/71 (12.7)	0.97 (0.47–2.01)	0.88 (0.42–1.84)	0.83 (0.40–1.75)	0.67 (0.31–1.45)
Purpose in life only	36/159 (22.6)	1.96 (1.29–2.99)	1.84 (1.19–2.82)	1.81 (1.17–2.78)	1.58 (1.02–2.47)
Neither	20/62 (32.3)	3.19 (1.81–5.63)	2.86 (1.60–5.11)	2.68 (1.49–4.81)	1.89 (1.01–3.55)

ADL, activities of daily living; CI, confidence interval; IADL, instrumental activities of daily living; OR, odds ratio.

^a^The number of people who developed an outcome (a decline in ADL or IADL) during follow-up.

^b^The number of people who had independent ADL or IADL at baseline.

Model 1: Unadjusted.

Model 2: Adjusted for age and gender.

Model 3: Adjusted for age, gender, income, BMI, alcohol intake, smoking, and the number of chronic diseases.

Model 4: In addition to Model 3, cognitive functioning and depression were included.

Table 4 shows the results from analyses stratified by age, income, cognitive functioning, and depression. Significant associations of having neither hobbies nor PIL with mortality did not vary depending on age, income, or the presence of depression but were influenced by the status of cognitive functioning. Significant associations of having PIL only were observed among all subjects except for those aged 65–74 years and those with intact cognitive functioning. Significant associations of having neither hobbies nor PIL with ADL were unchanged according to age, income, or the status of cognitive functioning but were affected by the presence of depression. Significant associations of having PIL only disappeared for subjects aged 65–74 years and for those with depression. Among subjects with high income, poor cognitive functioning, or depression, there was no association between having hobbies, having PIL, and a decline in IADL. Significant associations of having neither hobbies nor PIL were observed irrespective of age, and among subjects with low income or those with intact cognitive functioning. Significant associations of having PIL only were observed only for subjects with low income and for those without depression.

**Table 4.** Stratified analyses by age, income, cognitive functioning, and depression

	Mortality		ADL decline		IADL decline	
		
	*n*	Adjusted HR (95% CI)	*n*	Adjusted OR (95% CI)	*n*	Adjusted OR (95% CI)
**Among subjects aged 65–74 years^a^**						
Both	599	1.00	519	1.00	493	1.00
Hobbies only	50	0.82 (0.20–3.47)	33	2.44 (0.51–11.68)	32	1.08 (0.30–3.85)
Purpose in life only	94	2.03 (0.92–4.52)	71	0.90 (0.20–4.06)	65	1.65 (0.74–3.68)
Neither	54	2.45 (1.04–5.79)	27	11.59 (4.02–33.37)	22	5.12 (1.88–13.94)
**Among subjects aged ≥75 years^a^**						
Both	557	1.00	397	1.00	377	1.00
Hobbies only	87	1.37 (0.79–2.39)	48	2.82 (1.28–6.21)	39	0.82 (0.31–2.19)
Purpose in life only	209	1.62 (1.10–2.39)	107	3.14 (1.78–5.54)	94	1.71 (0.99–2.97)
Neither	203	2.47 (1.72–3.54)	52	2.80 (1.33–5.88)	40	2.31 (1.09–4.91)
**Among subjects with high income^b^**						
Both	752	1.00	600	1.00	570	1.00
Hobbies only	73	1.09 (0.47–2.53)	45	1.88 (0.61–5.82)	36	0.61 (0.18–2.09)
Purpose in life only	151	2.12 (1.31–3.43)	97	3.21 (1.60–6.44)	86	1.68 (0.93–3.04)
Neither	108	3.05 (1.94–4.80)	38	4.05 (1.63–10.10)	32	2.13 (0.92–4.92)
**Among subjects with low income^b^**						
Both	404	1.00	316	1.00	300	1.00
Hobbies only	64	1.96 (1.02–3.78)	36	3.27 (1.35–7.92)	35	1.12 (0.43–2.92)
Purpose in life only	152	2.06 (1.27–3.34)	81	2.76 (1.38–5.53)	73	2.03 (1.07–3.84)
Neither	149	3.73 (2.41–5.79)	41	4.73 (2.12–10.58)	30	3.44 (1.49–7.93)
**Among subjects with poor cognitive functioning^c^**						
Both	208	1.00	127	1.00	113	1.00
Hobbies only	61	1.66 (0.85–3.22)	21	0.75 (0.16–3.61)	19	0.75 (0.22–2.58)
Purpose in life only	119	2.41 (1.46–3.97)	45	2.42 (1.03–5.67)	38	1.62 (0.73–3.59)
Neither	185	3.26 (2.08–5.09)	34	4.02 (1.61–10.02)	21	1.70 (0.61–4.77)
**Among subjects with intact cognitive functioning^c^**						
Both	948	1.00	789	1.00	757	1.00
Hobbies only	76	0.90 (0.36–2.24)	60	4.58 (2.15–9.76)	52	0.76 (0.29–1.98)
Purpose in life only	184	1.34 (0.80–2.26)	133	3.48 (1.91–6.32)	121	1.68 (0.99–2.86)
Neither	72	1.22 (0.58–2.58)	45	4.23 (1.85–9.71)	41	2.91 (1.40–6.05)
**Among subjects with depression^c^**						
Both	251	1.00	156	1.00	147	1.00
Hobbies only	71	1.23 (0.64–2.38)	34	1.60 (0.62–4.12)	28	0.42 (0.13–1.37)
Purpose in life only	118	1.77 (1.09–2.87)	56	1.44 (0.63–3.30)	49	1.27 (0.59–2.73)
Neither	190	1.97 (1.30–3.01)	57	1.96 (0.89–4.29)	44	1.67 (0.77–3.60)
**Among subjects without depression^c^**						
Both	905	1.00	760	1.00	723	1.00
Hobbies only	66	1.42 (0.61–3.31)	47	2.06 (0.68–6.24)	43	0.96 (0.36–2.55)
Purpose in life only	185	1.88 (1.15–3.05)	122	3.94 (2.12–7.31)	110	1.90 (1.11–3.25)
Neither	67	5.71 (3.38–9.64)	22	5.03 (1.66–15.24)	18	2.77 (0.93–8.24)

ADL, activities of daily living; CI, confidence interval; HR, hazard ratio; IADL, instrumental activities of daily living; OR, odds ratio.

^a^Adjusted for age (continuous variables), gender, income, body mass index, alcohol intake, smoking, and the number of chronic diseases.

^b^Adjusted for age (65–74 vs ≥75 years), gender, body mass index, alcohol intake, smoking, and the number of chronic diseases.

^c^Adjusted for age (65–74 vs ≥75 years), gender, income, body mass index, alcohol intake, smoking, and the number of chronic diseases.

## DISCUSSION

In this prospective cohort study, we assessed the relationships of having hobbies and PIL with mortality and decline in ADL and IADL among community-dwelling elderly adults. We found that having neither hobbies nor PIL was associated with a significantly higher risk of not only mortality, but also decline in ADL and IADL, after adjusting for potential confounders, including age, income, cognitive functioning, and the presence of depression. Additionally, our findings suggest that having no hobbies may be more strongly linked to the risk of mortality and functional decline than lacking PIL.

There are several possible mechanisms to explain the observed relationships. First, hobbies can possibly reinforce neural networks and musculoskeletal abilities needed to keep independently functioning as a person’s physiological reserve capacity deteriorates as they age (the “use it or lose it” hypothesis).^3^^,^^28^ A prior study has shown that leisure activities, including hobbies, are associated with a slower rate of motor function decline.^29^ Second, a potential explanation for why having hobbies can prevent mortality and functional decline is an increase in physical activity due to hobbies. Prospective cohort studies of community-dwelling elderly adults have indicated that hobbies high in physical demand are more relevant to incident functional disability prevention than those involving a low level of physical activity.^30^^,^^31^ In our stratified analysis, the young elderly (aged 65–74 years) had no risk of mortality nor a decline in ADL and IADL, not only among the subjects with hobbies only but also those with PIL only. This result is in line with previous studies that showed that physical activity among the young elderly had less impact on survival^2^^,^^3^ and physical capacity^32^ compared to the older elderly. Third, the association of having hobbies with our outcomes might be mediated by an improvement in quality of life (QOL) derived from having hobbies. Prior studies found that the elderly with hobbies had a significantly higher level of QOL than those without hobbies^33^ and that better QOL was associated with reduced risk of mortality,^34^ difficulty in ADL,^35^ and IADL impairment.^36^ These mechanisms for the association of having hobbies with QOL and other outcomes are likely to be beneficial regardless of whether or not subjects have PIL, as subjects with hobbies but no PIL had no risk of a decline in ADL and IADL. Fourth, a prior population-based study investigated the association between the sense of having a life worth living (corresponding to PIL) and cause-specific mortality risk and found that increased mortality risk in those not having PIL had a significant risk of dying of cardiovascular disease.^13^ Other prospective cohort studies have reported that hypertension adversely affected ADL in persons free of diagnosed cardiovascular disease^37^ and that there was a significant relationship between the accumulation of cardiovascular risk factors based on medical examinations and a 5-year decline in IADL among community-dwelling elderly.^38^ Negative psychological state related to a lack of PIL could have the effect of changing an elderly person’s disease susceptibility due to the enhancement of sympathetic nervous system activity, decreasing heart rate variability, and increasing blood pressure,^39^ and this is often related to greater serum rates of both C-reactive protein and inflammatory cytokines and lower serum levels of high-density lipoprotein.^40^^–^^42^ Each of these factors are known to be cardiovascular disease risk markers.^13^ Taken together, a negative psychological condition due to the lack of PIL might promote the risk factor profile for cardiovascular disease, contributing to an increased risk of mortality and a decline in ADL and IADL. Last, reduced stress buffering is also considered a pathway to mortality and disability. Previous studies have shown that having PIL provides people with the ability to integrate stressful psychological events with minimal confusion.^43^^,^^44^ This ability decreases psychiatric/somatic symptoms that occur in stressful conditions,^43^ which contribute to a protective effect on mortality.^45^ A prospective cohort study reported that engagement in hobby organizations decreased the risk of incident functional disability in community-dwelling elderly and indicated that the prevention of functional decline may be partly explained by the stress-buffering protective effect of hobbies.^30^ Taken together, since having hobbies and PIL allows an individual to manage stressful situations better, they can shield elderly people from mortality and the functional decline associated with stressful experiences.

Our findings suggest that a lack of hobbies and PIL may be a cause of ADL and IADL decline, since it was a longitudinal study and no subjects had shown any decline when the follow-up began. However, we cannot completely exclude the possibility of reverse causation. A number of reports have shown that age, low socioeconomic status, cognitive impairment, and depression are predictive factors for disability in elderly people.^37^^,^^46^^–^^48^ To confirm the effects of these factors in relation to the association between having hobbies, having PIL, and ADL or IADL, we also conducted an analysis stratified by age, income, cognitive functioning, and depression. Our results suggest that age and income have little influence, but the status of cognitive functioning and the presence of depression modify the associations. These findings are consistent with those of previous studies of community-dwelling older persons that depressive symptoms affect social engagement, including hobbies^49^ and PIL (*ikigai*),^50^ and that cognitive impairment is associated with social disengagement^51^ and poorer sense of PIL.^52^ Therefore, attention should be paid to the fact that cognitive functioning and depression may modify the effects of our findings.

There are several limitations to our study. First, in the present study, PIL (*ikigai*) was assessed by a single, simple question. Although a single, simple question is useful for community-based health promotion and is the most commonly used assessment method in studies on “*ikigai*”,^12^^,^^13^^,^^15^^,^^53^^,^^54^ its validity and reliability have not been examined. The sense of “*ikigai*”, which is culturally defined as a subjective evaluation of well-being among Japanese, is often seen as an emotional concept in which a person can look back at how they have lived their lives, the joy and pride they have had, and the self-satisfaction it gives them.^53^ Recently, some researchers have developed scales consisting of multiple items to assess multidimensional aspects of “*ikigai*”.^55^^–^^57^ Therefore, our results should be confirmed using more recent tools. Second, our results may have been biased by the exclusion of subjects who did not provide the required data or did not return the questionnaire, as individuals aged 75 years and older and individuals with low income were more likely to be excluded subjects (eTable 1). Although we have no baseline data for the non-responders, it’s possible that they may have had poor or failing psychological situations or functional capacity that resulted in their discontinuation from the study. We hypothesize that those at high risk for mortality and disability may have been differentially excluded in this study. This may have resulted in an underestimation of the association of having hobbies and PIL with mortality, ADL, and IADL. Third, the hobbies and PIL among our study subjects may have changed—both positively and negatively—while they were in the follow-up period. Unfortunately, we had no access to such data. Last, we realize that some other unforeseen factors could have altered our observations. One example would be our lack of physical activity data. Because population-based surveys of the elderly have shown that those without participation in hobbies^10^ or without PIL^13^ are less likely to exercise compared with those with hobbies or PIL and that physical activity is associated with a reduced risk of mortality^2^^,^^3^ and disability,^58^ including physical activity data, might have changed the associations we observed.

In conclusion, we found that having hobbies and PIL affected not only the risk of mortality but also decline in ADL and IADL among community-dwelling elderly. Although we cannot rule out the possibility that having hobby activities and PIL may be affected by the status of cognitive functioning and the presence of depression, our findings suggest that health-promoting approaches, such as promoting engagement in hobbies and encouraging PIL, may be useful in preventing decline in ADL and IADL as well as reducing the risk of mortality.

## ONLINE ONLY MATERIAL

eTable 1. Basic attributes of subjects with or without valid responses at baseline and analyzed or excluded subjects at follow-up.
