# Supplementary material for: Relationship of Having Hobbies and a Purpose in Life With Mortality, Activities of Daily Living, and Instrumental Activities of Daily Living Among Community-Dwelling Elderly Adults
Source: J Epidemiol. 2016 Jul 5;26(7):361–70. doi: 10.2188/jea.JE20150153 (PMC4919481; doi:10.2188/jea.JE20150153)
Supplement: eTable 1. [file je-26-361-s001.pdf]

eTable 1. Basic attributes of subjects with or without valid responses at baseline and analyzed or excluded subjects at follow-up

Basic attributes at baseline	Baseline			Follow-up					
				ADL			IADL		
	Valid responses (n=1,853)	Invalid responses[a] (n=208)	*P* [b]	Analyzed subjects (n=1,254)	Excluded subjects[c] (n=302)	*P* [b]	Analyzed subjects (n=1,162)	Excluded subjects[c] (n=237)	*P* [b]
75 years of age and older	1,056 (57.0)	133 (63.9)	0.055	604 (48.2)	198 (65.6)	<0.001	550 (47.3)	144 (60.8)	<0.001
Male	783 (42.3)	75 (36.1)	0.088	522 (41.6)	146 (48.3)	0.106	454 (39.1)	109 (46.0)	0.001
Low income	769 (41.5)	83 (39.9)	0.711	474 (37.8)	138 (45.7)	<0.001	438 (37.7)	106 (44.7)	0.043

Data are given as n (%).

ADL, activities of daily living; IADL, instrumental activities of daily living.

[a] Subjects with invalid responses for ADL, IADL, hobbies, and/or a purpose in life

[b] Differences between the two groups were analyzed using Fisher's exact test.

[c] Subjects who did not participate in the follow-up survey or had invalid responses for ADL or IADL at follow-up
